# Vascular pythiosis of carotid artery with meningitis and cerebral septic emboli: A case report and literature review

**DOI:** 10.1016/j.mmcr.2018.05.003

**Published:** 2018-05-07

**Authors:** Maria Nina Chitasombat, Porkaew Petchkum, Suthas Horsirimanont, Pattana Sornmayura, Ariya Chindamporn, Theerapong Krajaejun

**Affiliations:** a Division of Infectious Disease, Department of Medicine, Faculty of Medicine, Ramathibodi Hospital, Mahidol University, Bangkok, Thailand; b Department of Surgery, Faculty of Medicine, Ramathibodi Hospital, Mahidol University, Bangkok, Thailand; c Department of Pathology, Faculty of Medicine, Ramathibodi Hospital, Mahidol University, Bangkok, Thailand; d Mycology Unit, Department of Microbiology, Faculty of Medicine, Chulalongkorn University, Bangkok, Thailand

**Keywords:** Pythiosis, *Pythium insidiosum*, Carotid artery, Meningitis, Brain

## Abstract

Vascular Pythiosis caused by *Pythium insiodiosum* rarely involves carotid artery. A case of concealed ruptured pseudoaneurysm of the carotid artery with neck abscesses, and cerebral septic emboli is described. Patient presented with large pulsatile neck mass that failed to response to surgery, antifungals and immunotherapeutic vaccine. Residual unresectable disease leads to death in the patient. Pythiosis should be considered as a differential diagnosis of head and neck infection.

## 1 Introduction

Human pythiosis is endemic in Thailand, tropical and subtropical area of the world known to cause disease in animal and human [1]. Pythiosis caused by *Pythium insidiosum*, Kingdom Straminipila, Phylum Oomycota known as aquatic fungi [1]. Pythiosis inhabits aquatic area, motile spore act as infective unit which attached to skin and penetrates to deeper layer resulted in several forms eg. skin, subcutaneous tissue, cornea, vascular, disseminated form [1], [2]. Risk factor of vascular or disseminated pythiosis included thalassemia, hemoglobinopathy, paroxysmal nocturnal hemoglobiuria, aplastic anemia, and leukemia [1]. Pythiosis is a disease that had several challenges in both diagnosis and treatment. Although nowadays, there are several new diagnostic methods such as polymerase chain reaction from direct specimen [3], immuno-histochemical staining [4], and serodiagnosis such as enzyme-linked immunosorbent assay [5], hemagglutination test [6], western blotting technique, serum immunodiffusion, immunochromatographic test*,* the gold standard remains tissue fungal culture which is a time consuming method [7], [8], [9], [10]. Early recognition of pythiosis and confirmed by rapid serodiagnosis could enhance the rapid clinical management for such life threatening disease. Pythiosis had several clinical presentations; vascular form is a life/limb threatening disease, commonly involved medium-to-large-sized arteries of lower extremities resulted in ascending blood vessel infection, aneurysm and thrombosis [1], [11]. Carotid vessel involvement is rare, only one report from literature review [12]. Radical surgery to achieve organism-free margin is the mainstay of successful treatment of vascular pythiosis [11]. However, this method is difficult to achieve for head/neck area. Treatment of unresectable disease with antifungal therapy alone had few successful outcome [1]. Immunotherapeutic vaccine; *P. insidiosum* antigen (PIA) vaccine demonstrate efficacy in an inoperable case [12]. Nowadays, PIA vaccine is used as an adjunctive treatment to combination of terbinafine and itraconazole for the disease with high morbidity and mortality [13]. In this report, we described the first case of concealed rupture of left carotid artery pseudoaneurysm associated para-pharyngeal abscesses with meningitis and cerebral septic emboli caused by *P. insidiosum* in an alcoholic patient.

## 2 Case

**Fig. 1:**
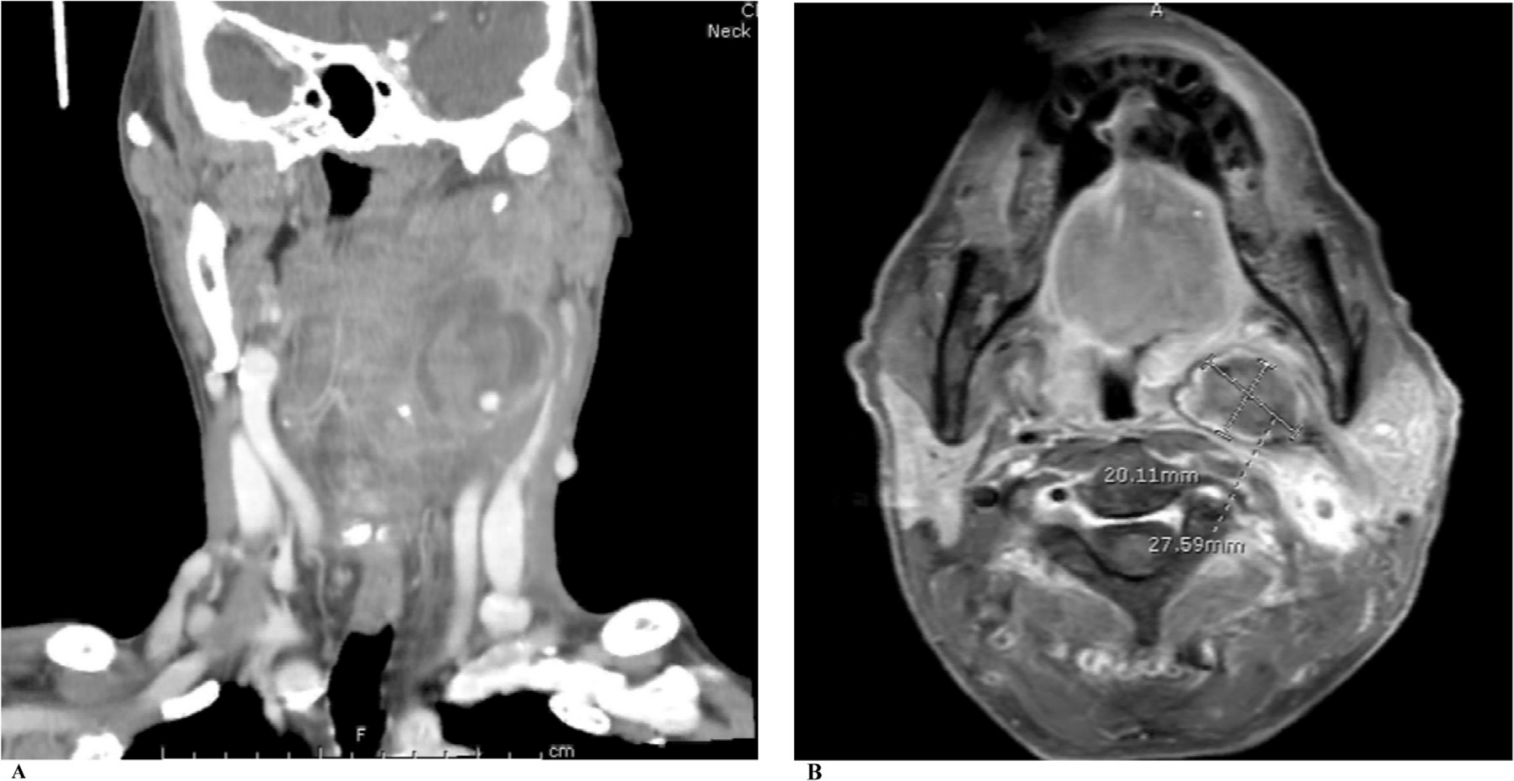
A. Computer tomography of the neck (coronal plain) showed a concealed ruptured of left external carotid artery aneurysm with surrounding hematoma at the medial aspect resulted in the narrowing of upper airway. B. Computer tomography of the neck (cross sectional plain).

**Fig. 2:**
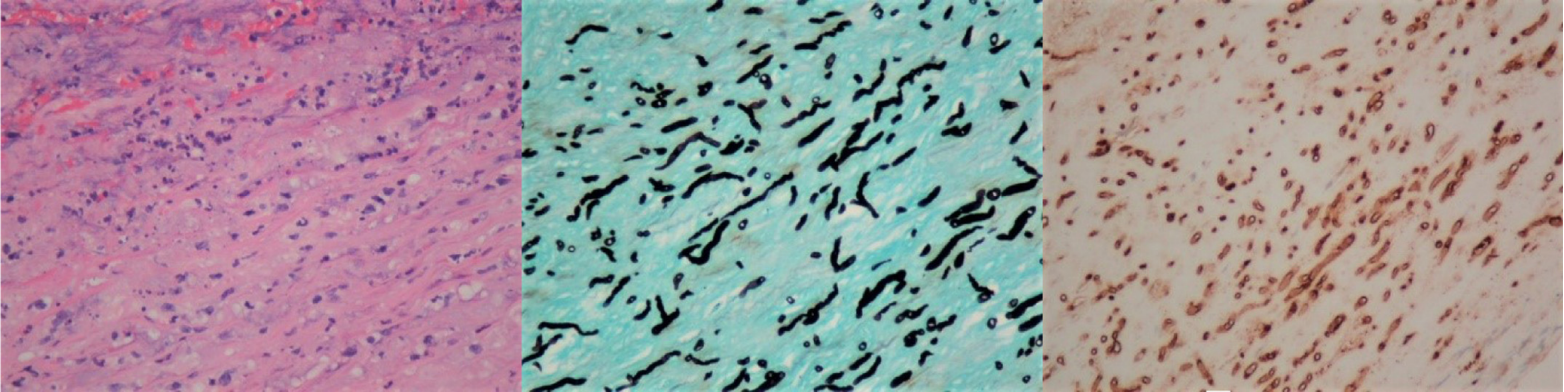
Histopathology of carotid vessel. Power photomicrograph: [400 × Hematoxylin and eosin stain] revealed acute suppurative inflammation. A. Histopathology of carotid vessel. Power photomicrograph: [400 × Gomori Methanamine Silver stain] revealed branching broad rare septate hyphae. B. Histopathology of carotid vessel. Power photomicrograph: [400 × Immunohistochemistry stain for *P. insidiosum* stain] revealed branching broad rare septate hyphae with positive stain.

**Fig. 3:**
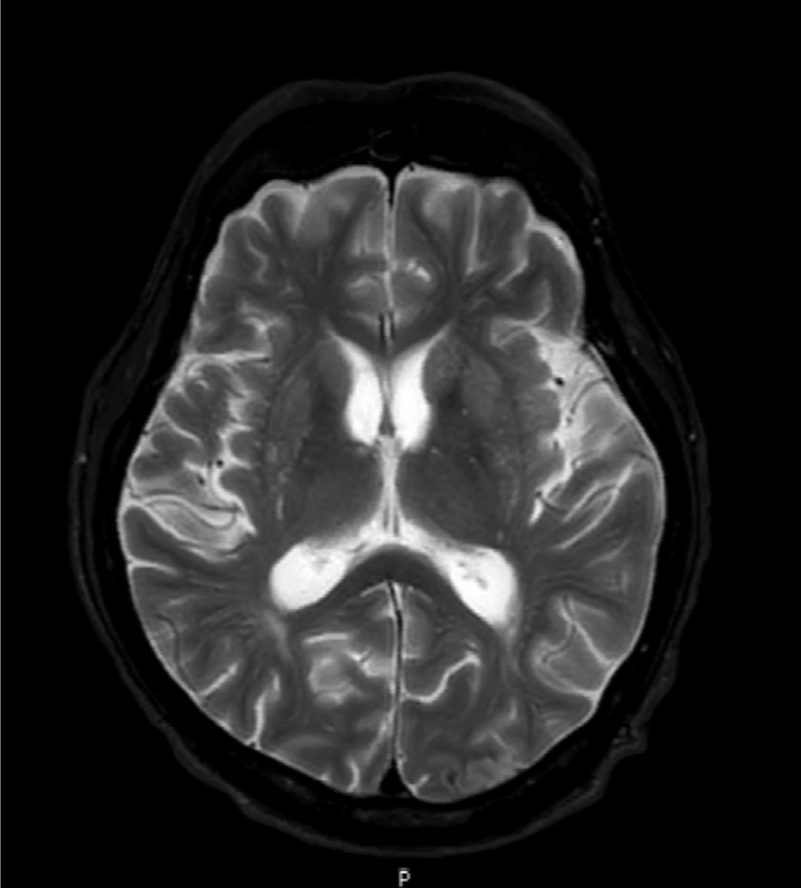
Magnetic resonance of the brain (T2FS) showed multifocal foci of restricted diffusion with internal microhemorrhage consistent with cerebral septic emboli and leptomeningeal enhancement at the left cerebral hemisphere.

A 57-year-old Thai man from Sa Kaeo, a province in the Eastern region of Thailand referred to a University Teaching Hospital in Bangkok due to swelling and pain at the left side of the neck for one month. One week prior he was admitted to the local hospital due to low-grade fever, difficulty swallowing and hoarseness. He received intravenous ceftriaxone and clindamycin for presumptive diagnosis of deep neck infection. He had history of hypertension treated with amlodipine 10 mg and enalapril 10 mg daily. He had habits of heavy alcohol drinking for 40 years, and smoking. He works at the department of fisheries. He swam, cleaned fish pond and mowed the lawn. Upon admission (*day 0*), the patient's body weight was 52 kg, body mass index was 19.7 kg/m^2^. His vital signs were as follows: body temperature, 38.7 °C; blood pressure, 170/100 mmHg, pulse rate, 100 beats/min; respiratory rate, 24 breaths/min. On physical examination, mild pale conjuctivae, anicteric sclerae. The neck exam revealed pulsatile left neck mass size 5 × 5 cm in diameters, mild tender on palpation, no sign of inflammation. No limitation of neck movement. Oropharyngeal exam revealed bulging of left posterior pharyngeal wall and tonsil enlargement causing the narrowing of upper airway. Thyroid gland was not enlarged. Other exams included neurological exam were normal. Skin exam revealed multiple ill-defined scaly mild erythematous patches on both legs and dystrophic nails. Initial laboratory results showed anemia with hemoglobin concentration of 9.6 g/L and Hematocrit of 28%, MCV of 75 fl, white blood cell count of 6800 cells/mm^3^ with 80% neutrophil 7.7% lymphocytes, platelet count of 574,000 cells/mm^3^. Hemoglobin typing was normal (HbF 0.2% HbA2 2.9% HbA 85.9%; HbA2A). Liver function test showed AST 87 U/L, ALT 97 U/L, ALP 127 U/L, GGT 936 U/L, TB 0.3 mg/dl, DB 0.1 mg/dl, TP 81.2 g/L, Alb 28.7 g/L. Fasting glucose of 95 mg/dL, HbA1C of 4.74%, BUN 14 mg/dL, Cr 1.05 mg/dL, Anti-HIV test was negative. Viral hepatitis profile were negative. His chest X-ray was normal. He was diagnosed with anemia of chronic disease, alcoholic hepatitis, and xerotic eczema. Computer tomography of the neck showed a concealed ruptured of left external carotid artery 0.9 × 1.9 cm in size with surrounding hematoma (3.6 × 3.6 ×5.8 cm) at medial aspect of an aneurysm resulted in narrowing of the upper airway (Fig. 1A, B). Urgent surgical exploration on *day 0* revealed severe adhesion around pseudoaneurysm (size 5 × 6 cm) confined around common carotid artery, carotid bifurcation, extended to the angle of mandible. The diameter of pseudoaneurysm neck was one cm, located at medial wall of common carotid artery just distal to carotid bifurcation. External carotid artery was obliterated. Angiogram and balloon occlusion was performed at the left common carotid artery. External carotid artery and internal carotid artery were ligated at the arterial stump just beneath the angle of mandible. Pseudoaneurysm was resected and internal content show pus and clot. Surgical margins were not free in gross section. The pus was sent for bacterial culture. Blood agar plate revealed rare growth of whitish colony, direct exam from the colony revealed broad rare septate fungal hyphae. Infectious disease was consulted on *day 5* of admission. Serum antibodies to pythium antigen using an in-house rapid immunochromatographic test were positive on *day 5*. Sabouraud's glucose agar (SGA) grew fungal colony which identified as *P. insidiosum* by the induction of motile zoospore and confirmed by fungal broad-range 18S rDNA gene polymerase chain reaction. Pathology of carotid artery revealed acute suppurative inflammation (Fig. 2) with branching broad rare septate hyphae demonstrated by Gomori Methanamine Silver stain and Periodic acid-Shiff stain (Fig. 2A) Immunohistochemistry stain for *P. insidiosum* was positive (Fig. 2B). Medical therapy with oral itraconazole 200 mg oral twice daily combined with terbinafine 250 mg oral twice daily were started on *day 5*. Adjunctive immunotherapy with subcutaneous injection of PIA vaccine 500 microliter (4 mg/ml) was given on *day 6* and *18* of admission. Day 5, Computer tomography of the aorta shows atherosclerotic change without aneurysm or dissection. Postoperatively, physical exams revealed narrowing of upper airway, hypoglossal nerve palsy on the left side without motor deficit. Neurology was consulted on *day 6*. He was diagnosed with hypoglossal nerve palsy secondary to compression of carotid artery aneurysm. On *Day 6*, MRI and MRA of the brain revealed pseudoaneurysm of carotid artery at left carotid-parapharyngeal spaces (2.8 × 2.0 × 3.1 cm) associated with extensive inflammation of the surrounding soft tissue resulting in mild narrowing of upper airway. Left common carotid artery was occluded along the origin to the cavernous part of left internal carotid artery with the evidence of wall enhancement. Multifocal cerebritis consistent with cerebral septic emboli and leptomeningeal enhancement at the left cerebral hemisphere (Fig. 3). The patient underwent second exploration of the left neck on *day 9* aiming to remove the residual infected necrotic tissue. Operative findings revealed pus with necrotic soft tissue extended to parapharyngeal space, however artery cannot be defined. The radical neck dissection could not be performed due to the morbidity outweigh the possibility of the cure. Tissue specimen revealed identical findings with the first operation. On **day 6**, the dosage of terbinafine was increased to 250 mg three times daily, itraconazole was continued. The patient and family decided for palliative care, no aggressive treatment. He was discharged on *day 19* of hospitalization. On *day 29* after discharge, upon an outpatient visit, his family mentioned that he developed progressive right hemiparesis over the two days after discharge. Physical exams revealed healed surgical wound of the left neck, narrowing of upper airway, neurological exams revealed global aphasia, right facial palsy (upper motor neuron), motor power grade I on the right side. His family denied further investigation. He continued to take combination of oral terbinafine, itraconazole and PIA vaccine. On *day 49*, upon an outpatient visit, he had flaccid hemiparesis on the right side without other deficit. He received fourth dose of PIA vaccine and continued oral itraconazole and terbinafine. On *day 82*, he expired at a local hospital due to complication of diseases.

## 3 Discussion

We report the first case of concealed rupture of large carotid artery pseudoaneurysm with parapharyngeal abscess, meningitis and cerebral septic emboli caused by *P. insidiosum* in an alcoholic patient. Unfortunately our patient had an unresectable disease which fail to respond to medical and immunotherapeutic vaccine. The acquisition of pythiosis in our patient most likely due to occupational exposure to aquatic habitats by swimming in the pond and contracted the disease through motile spore which penetrated subcutaneous tissue extended to vessels resulted in arteritis and mycotic aneurysm [1], [2]. The delay of time from initial symptoms to the diagnosis contributed to the advance stage of disease upon presentation. Early diagnosis in our patient was rather difficult due to several unusual characteristics which did not fit with typical pattern of recognition. Firstly, host factor, patient had alcoholism, malnourisment, and anemia of chronic disease which has never been report as risk factor for vascular pythiosis, unlike hemoglobinopathy which was described in almost all Thai patient with vascular or disseminated pythiosis [1]. Secondly, the site of involvement; vascular pythiosis usually involve lower extremitities rather than head/neck region or carotid vessel which is extremely rare [12]. The diagnosis was eventually made after the surgery. In terms of treatment, the mainstay of vascular pythiosis is radical surgery to achieve the organism free margin which is not feasible in our patient with intracranial vessel involvement [11]. Treatment with antifungal therapy alone had few successful outcome, particularly in an unresectable disease [1]. Most antifungals are not active against *P.insidiosum* due to the lack of ergosterol [14]. Among all antifungals, itraconazole and terbinafine had the lowest MIC of 0.125–4, 0.03–4 μg/ml respectively against clinical isolates of *P. insidiosum* [15], [16], [17]. Newer triazole such as voriconazole and posaconazole had MIC of 1, 0.125 μg/ml, respectively [17]. Combination of itraconazole and terbinafine had synergistic activity in vitro, however among Thai *P. insidiosum* strains were absent [15], [16]. The plausible explanations are the evidences of clade differences among *P. insidiosum* strain in each continent which contributed to variable in vitro susceptibilities among strains eg. Brazil vs. Thai isolates [14], [18], [19]. However, interpretation of susceptibility should be done with precaution due to there are several different methods of antifungal susceptibility determination with no standardized assay [14], [15]. New methods of in vitro susceptibilities by the radial growth assay showed that terbinafine was more inhibitory than itraconazole against the *P. insidiosum* with a dose dependent matter [14]. *P. insidiosum* isolates were sensitive to the antifungal agents only at concentrations that are difficult or impossible to achieve in vivo (> 8 mg/liter) which explain the clinical resistance of the drugs in the treatment of human pythiosis [14]. Combination of oral itraconazole and terbinafine remains the most commonly used regimen for treatment of pythiosis in Thailand, as used in our patient [14].

Immunotherapeutic vaccine; *P. insidiosum* vaccine (PIA) which derived from endoplasmic and secretory antigens of *P. insidiosum* [1] also being used successfully as adjunctive treatment to combination of itraconazole and terbinafine in an unresectable case; unresponsive deep tissue infection invading the carotid artery [12]. *P. insidiosum* vaccine demonstrate safety profile, however the efficacy of pythium vaccine still inconclusive [13], [15]. PIA vaccine has been used as immunotherapy among patient with inoperable vascular pythiosis with some successful outcome (cured 5/12 cases)[13]. Nowadays in Thailand*,* PIA vaccine was given as adjunctive treatment [1], [11], [15]. Our patient received four doses of PIA vaccine.

Pythiosis involved head and neck area, as our patient is extremely rare. Herein, we summarizes clinical manifestations, diagnosis, treatment and outcome of the unresectable disease of pythiosis involved head and neck region in Table 1 [1], [12], [16], [17], [20]. All adult cases with carotid vessel/brain involvement died. The only survived Thai pediatric patient (case no. 1) with unresectable disease had dramatic response to PIA vaccine, after discontinuation of medical therapy due to failure [12]. As clinical observation among survived pediatric patients with unresectable disease, granulocyte-monocyte colony-stimulating factor and interferon gamma were given in addition to PIA vaccine. This may stimulate the shift of immune response from Th2 to Th1 which enhance cytotoxic T cell killing of organism [13]. The first patient (case no. 1) whom solely received immunotherapy for unresponsive deep tissue invasive disease had large wheal reaction at the site of vaccine injection [12]. Case no 3. had swelling of the air passage, respiratory distress that required intubation, corticosteroid treatment after received the third dose of PIA vaccine which lead to discontinuation [17]. Our case had absent skin reaction, which we suspected that patient may have certain degree of cellular immunity impairment due to alcoholism and malnutrition.

**Table 1:** Characteristics of patients with pythiosis involve head and neck area.

						Treatment			Outcome (years)
Case no. (ref)	Case Age (Y)/ Sex	Underlying disease	Clinical manifestations	Vascular involvement	Diagnosis	Surgery	Drugs (duration, months)	PIA immunotherapy (day, reaction)	
1. [12]	14/M	β Thal/HbE disease post splenectomy	- Severe headache and soft-tissue swelling at the occiput - Extensive facial, bilateral retromolar cellulitis, facial-palsy	External carotid artery aneusysm, and stenosis of the internal carotid artery (Left side)	- Mandibular abscesses culture isolating *P. insidiosum* - Pathology of vessel wall showed fungal hyphae on silver stain - Serology (ELISA method) positive titers of 1:6,400 (before vaccination)	1^st^ Surgical drainage of the abscesses	- Amphotericin B, saturated potassium iodides ketoconazole (three months) Two courses of GM-CSF	Initiation after three months of failure to medical and surgical treatment	Survived. Follow up to 2 year. MRA at 1 year showed normal carotid artery with complete occlusion of external carotid artery
						2^nd^ Surgical exploration of parapharynx and masseteric space, removal of lymph node and great auricular nerve 3^rd^ Surgical resection of the aneurysm			
								D0 0.1 ml SC (wheal and flare reaction up to 11 cm at the injection site)	
								D14 0.1 ml SC (wheal reaction)	
								D28 0.1 ml SC (no reaction)	
2. [16]	2/M	None	- Preseptal cellulitis, maxillary sinusitis with parapharyngeal and retropharyngeal inflammation	None	Tissue culture isolating *P. insidiosum*	- Surgical biopsy of the affected tissue - Gastrostomy for feeding	Combination of oral terbinafine and itraconazole (one year)	None	Survived. Follow up time up to 1.5 year
3. [17]	10/F	None	- Rapidly progressing necrotizing orbital and facial infection. - Blindness, bilateral facial nerve palsies	None	- Orbital tissue biopsy pathology; GMS stain, culture, and PCR - Serology (enzyme-linked immunosorbent assay)	- Orbital biopsy - Left eye enucleation (at one year) - Multiple reconstructive surgeries	- Combination of oral itraconazole and terbinafine then posaconazole /voriconazole - GM-CSF and interferon-γ after vaccine discontinuation	D0 0.1 ml ID of extract at 1:10 dilution and 1:2 dilutions.	Survived. Follow up time up to at least 8 years
								D 2, 0.1 mL full-strength extract ID-(no reaction)	
								D3 0.5 mL SC (Massive facial swelling, ARDS, steroid treatment)	
4. [1]	44/M	Paroxysmal nocturnal hemoglobinuria	Orbital cellulitis with acute rhinosinusitis	NA	Serology (immunodiffusion method)	Surgical drainage	NA	None	Died (no autopsy)
5. [1]	26/F	Thallassemia	- Sudden onset of severe left-side headache. - CT brain showed hemorrhagic mass in the frontal brain area	NA	Brain tissue culture isolating *P. insidiosum*	Emergency craniotomy	NA	None	Died (no autopsy)
6. [20]	27/M	β Thal/HbE disease with secondary hemochromatosis	- Toothache at the left upper molars, nasal congestion, occipital headache and seizure - MRI brain showed Brain abscess size 5x6 cm at left cerebral hemisphere with satellite lesions	Multiple aneurysms, arterial dissection at left common and internal carotid arteries	- Serology (enzyme-linked immunosorbant assay, immunodiffusion and Western blot) - Pathology of carotid artery aneurysm-Wright’s stains. - Brain tissue culture (autopsy) isolating *P. insidiosum* confirmed by PCR	Double carotid stents	Amphotericin B, then combination of oral itraconazole and terbinafine	D0 SC (no skin reaction)	Died from brain herniation.
7. This report	57/M	Anemia of chronic disease, Alcoholism,HT	- Swollen of left neck - Concealed rupture of large infected pseudoaneurysm of left carotid artery associated para-pharyngeal abscess with meningitis and cerebral septic emboli	Carotid artery (left) with cerebral vessels	- Serology (immuno chromatographic test - Pathology of carotid artery aneurysm demonstrated by GMS stain - Vessel tissue isolating *P. insidiosum* from culture	1^st^ Resection of left external carotid artery aneusysm, ligation part of internal and external carotid artery	Combination of oral itraconazole and terbinafine	D0 1 ml SC 500 microliter (2mg/ml)	Died (no autopsy)
								D 13 1 ml SC	
								D 24 1 ml SC	
								D41 1 ml SC (no skin reaction)	
						2^nd^ Surgical debridement			

M:male, F: female, PIA: *P. insidiosum* antigen immunotherapy, β Thal/HbE: Beta-Thallassemia/Hemoglobin E, *ELISA*: enzyme-linked immunosorbent assay, GM-CSF: granulocyte-monocyte colony stimulating factor, SC: subcutaneous, MRA: magnetic resonance angiogram, GMS: Gomori Methanamine Silver, PCR: polymerase chain reaction, ID: intradermal, ARDS: adult respiratory distress syndrome, NA: not available, CT: computerized tomography, MRI: magnetic resonance imaging

Medical treatment with combination of itraconazole and terbinafine for 1.5 year alone achieved a successful outcome in one patient (case no. 2) with an inoperable deep tissue infection without vascular involvement [16]. The in vitro susceptibilities of *P. insidiosum* isolates revealed itraconazole MIC of 0.125 μg/ml, terbinafine MIC of 0.5 μg/ml; with an evidence of synergy which may explain the good clinical response in the patient [16].

Future research focus on evaluation of the cytokine response, the shift of humeral to cytotoxic immunity, cytotoxic T cell response after treatment with PIA vaccine in pediatric and adult patient may be able to determine the efficacy of vaccine. The correlation of skin reaction with the response to vaccine therapy should be assessed along with the treatment outcome. Customize and titration of the PIA vaccine dosage should be further evaluated to determine the vaccine efficacy.

In conclusion, pythiosis of the head/neck region is a rare life-threatening infection which can lead to carotid and intracranial arteritis, cerebral septic emboli, stroke and brain abscess. Radical surgery which is the primary curative measure is difficult to achieve for head/neck and intracranial lesions. Medical treatment and immunotherapy has achieved a success outcome in few patients with unresectable disease. Pythiosis should be in the differential diagnosis of carotid arteritis, necrotizing cellulitis of head and neck region among patient who had exposure to swampy area.
